# Tuberculosis of the knee in an immunocompetent adult: a case report

**DOI:** 10.1099/acmi.0.001095.v4

**Published:** 2026-09-03

**Authors:** R. Bounhir, I. Habboubat, E. Benaissa, Y. Ben Lahlou, M. Chadli

**Affiliations:** 1 Bacteriology Laboratory, Mohammed V Military Training Hospital, Rabat, Morocco; 2 Faculty of Medicine and Pharmacy, Rabat, Morocco

**Keywords:** GeneXpert, knee, tuberculosis

## Abstract

Osteoarticular tuberculosis (TB) is a rare but serious form of extrapulmonary TB that often presents with nonspecific symptoms, leading to delayed diagnosis and an increased risk of joint destruction. We report a case of knee TB in an immunocompetent patient, underscoring the diagnostic challenges and emphasizing the role of molecular techniques in the early management of this uncommon form of TB.

## Data Summary

No data were reused or generated in this study.

## Introduction

Tuberculosis (TB) is an infectious disease caused by *Mycobacterium tuberculosis* (Koch’s bacillus), which primarily affects the lungs [1]. Its airborne transmission and high virulence make it a major global public health concern [1].

It occurs in every part of the world and is particularly prevalent in immunosuppressed individuals [1]. It remains highly endemic in the World Health Organization (WHO) South-East Asia, African and Western Pacific Regions [1].

Osteoarticular tuberculosis (OA TB) is rare. It represents 2 to 5% of all TB cases [2] and 9 to 20% of extrapulmonary TB cases [3]. The spine is the most commonly affected site, followed by large weight-bearing joints such as the hip and knee [4].

Its diagnosis is based on a combination of clinical, radiological, biological and histopathological findings [5]. However, a diagnostic delay of 5 to 47 months after symptom onset has been reported, mainly due to nonspecific clinical features and insidious progression [3], thereby threatening joint function.

Microbiological testing plays an essential role in the diagnosis. Conventional methods, such as smear microscopy and culture, are essential; however, their sensitivity is low and results often take a long time [2]. Therefore, the WHO recommends rapid molecular tests, such as GeneXpert MTB/RIF Ultra, an automated rapid test that detects *M. tuberculosis* complex DNA and rifampicin resistance [1]. However, its use is only validated for sputum samples, and sensitivity may be reduced in extrapulmonary specimens [6].

This case illustrates the added value of molecular testing in overcoming diagnostic delays, highlighting its role in facilitating timely diagnosis and management. It also underscores the importance of considering TB even in immunocompetent patients and of systematically investigating potential associated pulmonary involvement.

## Case presentation

We report a 29-year-old immunocompetent male with no significant past medical history, presenting to our hospital in Morocco, which falls within the WHO Eastern Mediterranean Region, where TB remains highly prevalent [7]. The patient presented with a progressively painful swelling of the left knee, evolving over the past 2 months. Two weeks after symptom onset, the left knee effusion was aspirated by the referring physician, and the sample was submitted for routine bacterial culture targeting common pyogenic organisms. The culture subsequently remained sterile. Empirical treatment with oral amoxicillin/clavulanic acid 1 g/125 mg three times daily for 10 days was initiated immediately after the procedure. Despite completing the antibiotic course, the swelling and functional impairment continued to worsen, prompting consultation at our hospital ~1 month after finishing the treatment.

**Fig. 1.**
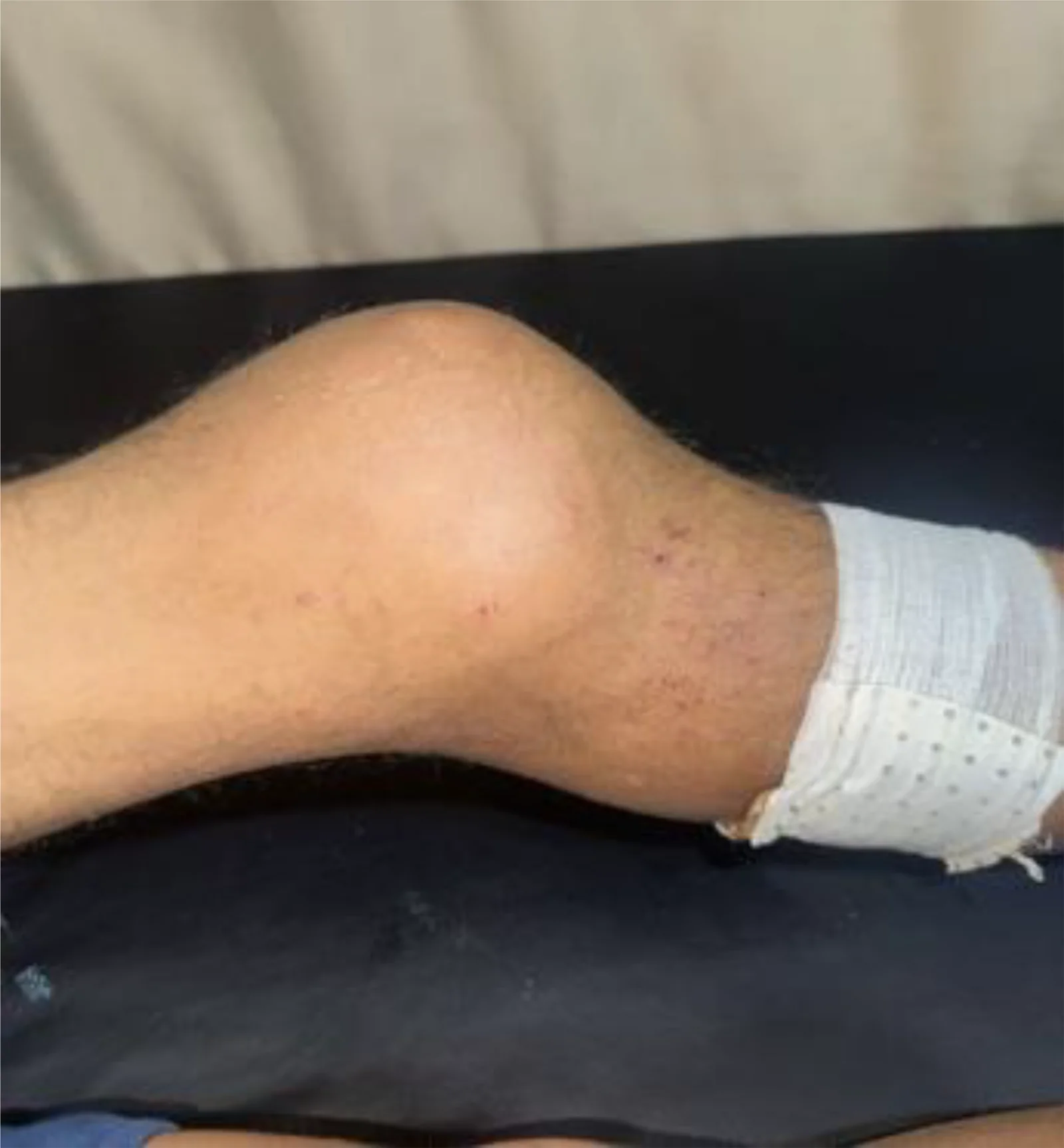
Swelling of the left knee, with a flexed posture.

On admission, the patient was stable and afebrile, reporting an unexplained weight loss of ~7 kg over the past 2 months. Physical examination revealed a painful swelling of the left knee without overt inflammatory signs, associated with a flexion contracture and markedly limited range of motion (Fig. 1).

Vascular, neurological and respiratory examinations were normal.

The blood tests showed a slight elevation in C-reactive protein levels of 36.5 mg l^−1^ (normal range <5 mg l^−1^), with a normal leucocyte count of 7,500 µl^−1^ (normal range: 4,000–11,000 µl^−1^). Serological tests for hepatitis B, syphilis and human immunodeficiency virus (HIV) were negative, while interferon-gamma release assay was positive.

**Fig. 2.**
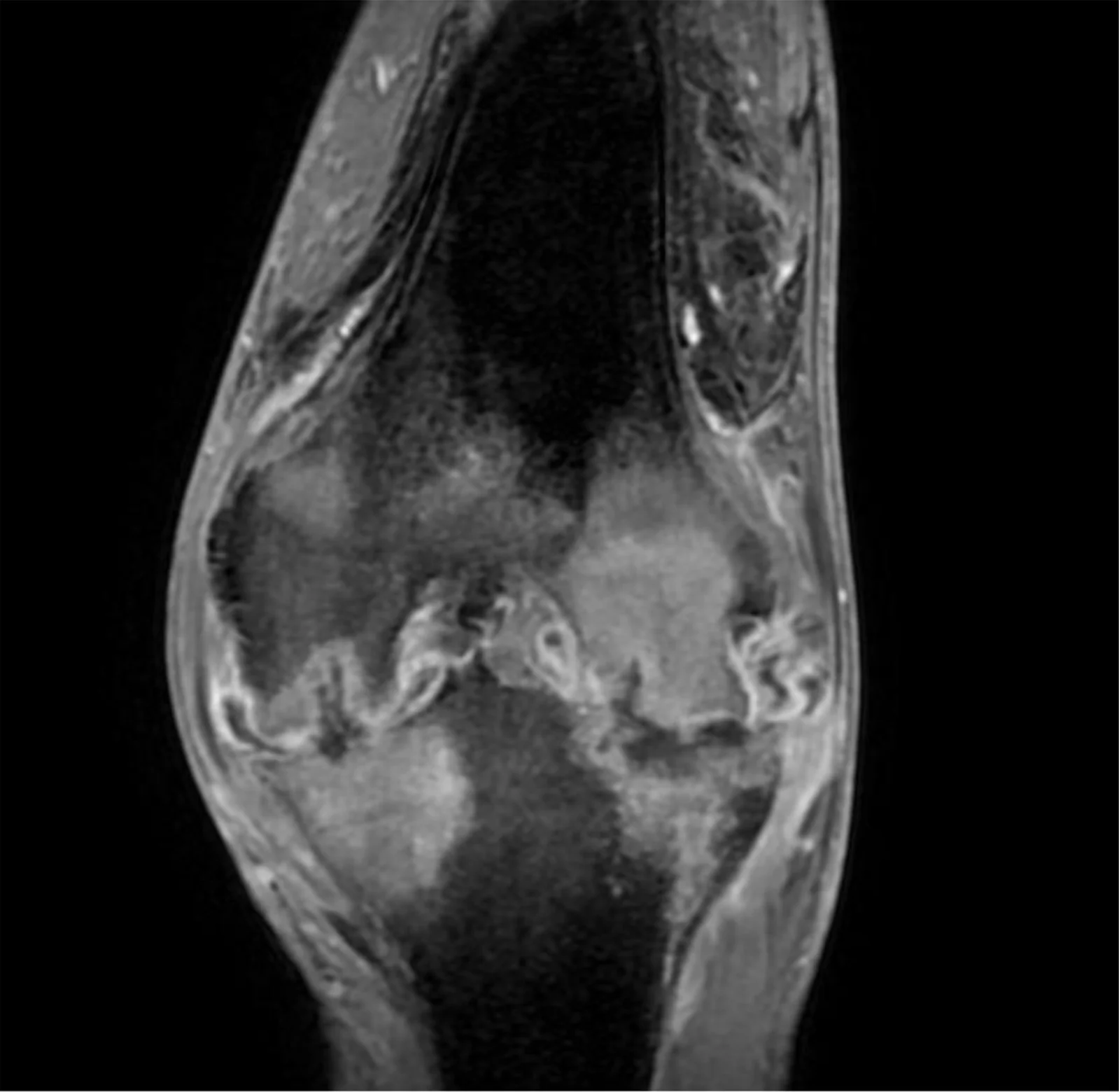
Coronal, post-contrast T1-weighted MRI of the left knee showing joint space narrowing with bone erosion of the tibial and femoral articular surfaces, marked bone marrow oedema and synovial enhancement.

**Fig. 3.**
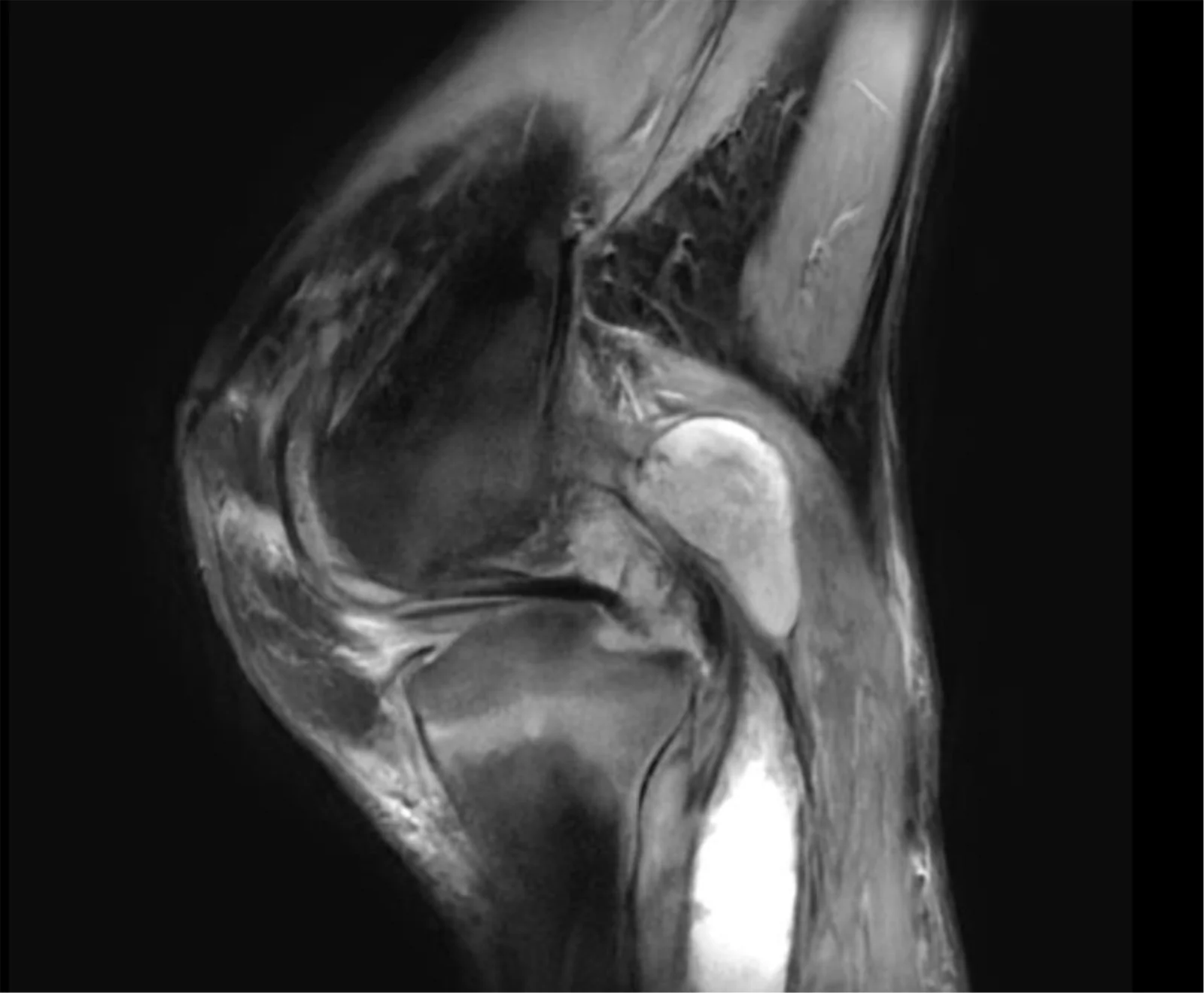
Sagittal proton density with fat saturation showing popliteal fossa collection.

Radiography and computed tomography (CT) scan of the left knee demonstrated joint space narrowing, cortical irregularities and subchondral erosions. Further assessment with magnetic resonance imaging (MRI) revealed diffuse bone marrow oedema, synovitis and two abscess collections in the posterior aspect of the knee (Figs 2 and 3).

In light of these findings, the patient underwent surgical drainage and lavage for abscess evacuation. During the procedure, three specimens were collected for laboratory analysis: synovial fluid, drainage fluid and bone tissue.

Comprehensive microbiological studies were performed, including Gram staining, aerobic and anaerobic cultures. Gram stain showed minimal to no inflammatory cells, with naked macrophage nuclei observed in the synovial fluid. No bacteria were detected.

Aerobic cultures were performed on Columbia agar with 5% sheep blood, chocolate agar and Brain–Heart Infusion broth for enrichment, which was then subcultured onto blood agar. Incubation was conducted aerobically at 37 °C for 18–24 h.

Anaerobic cultures were performed on Schaedler agar and Columbia blood agar supplemented with nalidixic acid and colistin, using an anaerobic jar (Oxoid) and an anaerobic gas-generating system (Oxoid AnaeroGen). Incubation was performed anaerobically at 37 °C for 48 h.

Culture plates were monitored every 24 h for aerobes and every 48 h for anaerobes. After 15 days of incubation, all cultures remained sterile.

Given the sterile cultures and chronic inflammatory findings, a specific infection such as TB was suspected. Therefore, targeted testing for *M. tuberculosis* complex was conducted on bone tissue using both conventional methods (Ziehl–Neelsen, auramine staining and culture) and molecular testing. Ziehl–Neelsen and auramine stains were negative, but PCR analysis using GeneXpert MTB/RIF Ultra detected *M. tuberculosis* complex at a low bacterial load, with no evidence of rifampicin resistance, confirming the diagnosis of knee TB about 5 days after admission.

Culture in the Mycobacteria Growth Indicator Tube medium became positive after 25 days, and culture on Löwenstein–Jensen medium became positive after 31 days of incubation.

Drug susceptibility testing using the HAIN line probe assay did not detect resistance to isoniazid and rifampicin.

Histological examination of the synovial and bone biopsies revealed necrotizing granulomatous inflammation with no evidence of malignancy.

**Fig. 4.**
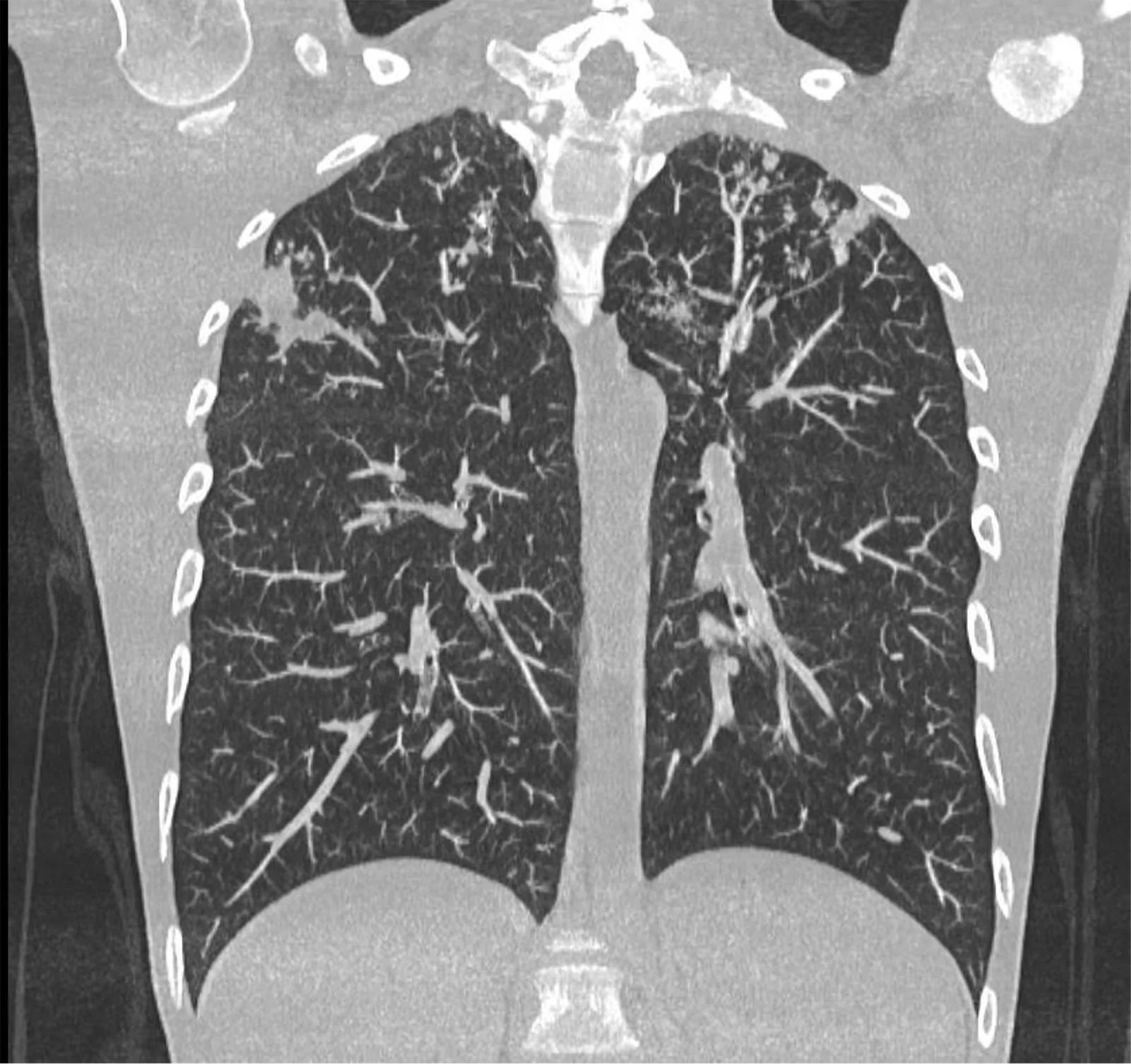
Coronal CT scan of the chest in parenchymal window with maximum intensity projection reconstruction: multiple bilateral micronodules in the upper lobes with a branching distribution, giving a ‘tree-in-bud’ appearance.

**Fig. 5.**
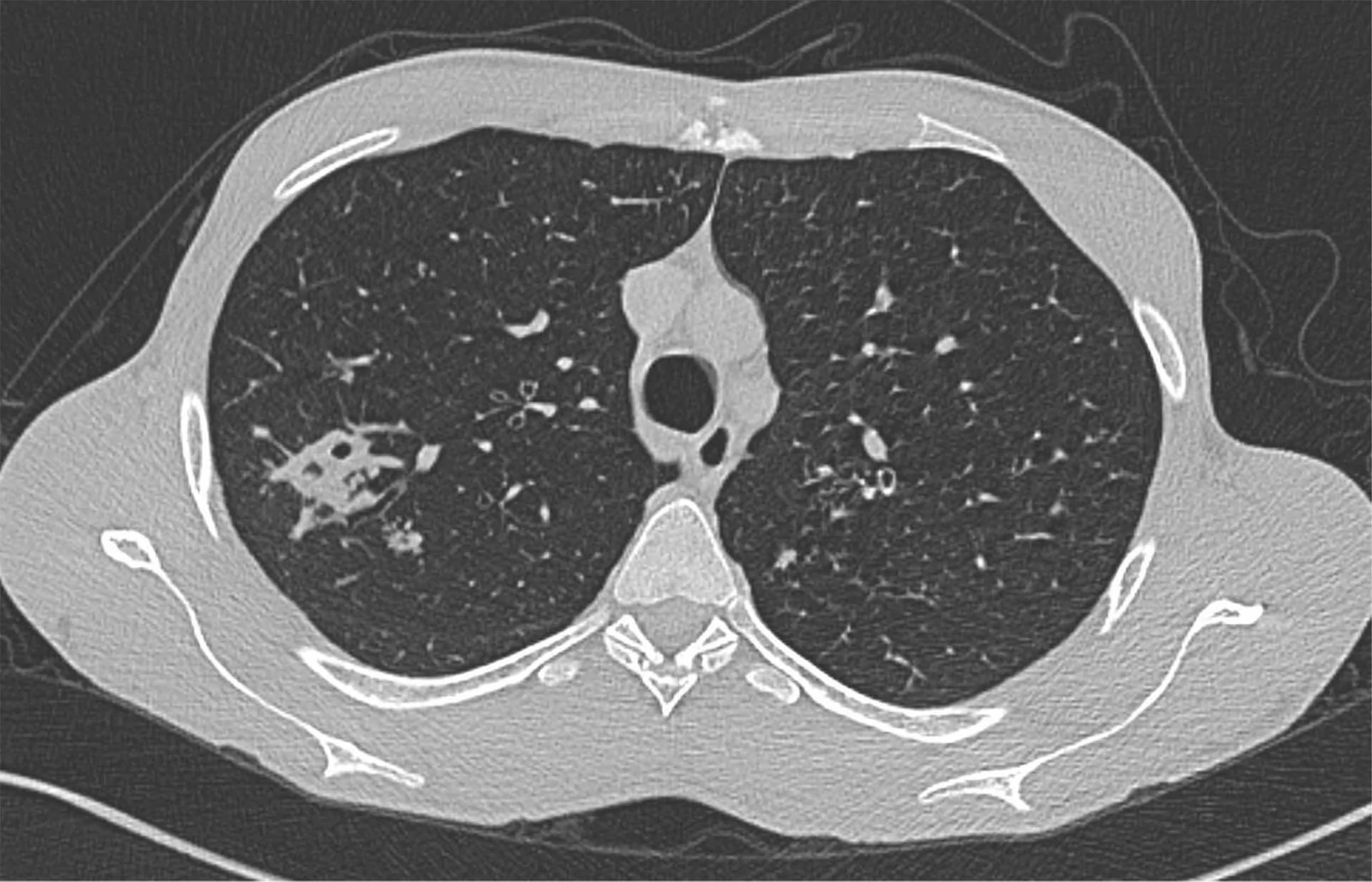
Axial CT scan of the chest showing cavitated nodule in the dorsal segment of the right upper lobe, surrounded by a few micronodules.

As part of the extrapulmonary TB workup, a thoracic CT scan was performed. It revealed an excavated nodule in the dorsal segment of the right upper lobe, along with multiple bilateral micronodules in the upper lobes, distributed in a branching pattern consistent with a ‘tree-in-bud’ appearance (Figs 4 and 5).

In parallel, GeneXpert MTB/RIF Ultra was performed on bronchial aspiration, confirming the presence of *M. tuberculosis* complex at low bacterial load without rifampicin resistance. Based on this result, a diagnosis of knee TB associated with pulmonary TB was established. The patient was transferred to the respiratory department and immediately started on anti-tuberculous therapy with isoniazid, rifampicin, pyrazinamide and ethambutol, without waiting for culture results. The treatment led to significant clinical, biological and radiological improvement.

## Discussion

OA TB is a rare manifestation of extrapulmonary TB, representing 9 to 20% of extrapulmonary TB cases [3]. The incidence is higher in regions where TB is endemic, particularly among patients with immunosuppression (HIV, diabetes and chronic renal failure) [3]. However, it can also occur in individuals without predisposing factors, as illustrated in our patient.

In addition to these epidemiological trends, the age distribution of OA TB also varies by region. In endemic areas, it predominantly affects young adults between 20 and 40 years of age, whereas in nonendemic regions it is more commonly observed in individuals over 55 years old [4]. In our case, the patient was 29 years old, which aligns with the typical demographic reported in endemic regions.

OA TB generally develops through haematogenous dissemination of *M. tuberculosis* complex from a primary site, often pulmonary or lymphatic. The infection may originate in bone or the synovial membrane, with rapid spread between them [8]. A cold abscess may form due to a pronounced exudative reaction, extending through the periosteum and surrounding tissues and leading to joint effusions or soft tissue collections [8].

Our patient exhibited the characteristic features of knee TB, including chronic swelling and functional impairment but did not present with respiratory symptoms. This highlights the importance of actively screening for pulmonary TB and considering TB even in immunocompetent patients, particularly in endemic regions. Early imaging, combined with prompt molecular testing such as GeneXpert, can enable rapid diagnosis and initiation of treatment, potentially improving functional outcomes.

Acid-fast bacillus smear microscopy and mycobacterial culture continue to play a major role in TB diagnosis but they have some limitations. Smear microscopy is rapid and cost-effective; however, its sensitivity is low, and its specificity is limited, as it cannot differentiate *M. tuberculosis* complex from nontuberculous mycobacteria [9]. Although culture is valuable, it is time-consuming and requires specialized infrastructure and technical expertise. Given these limitations, the WHO recommends the use of rapid molecular diagnostic methods [10].

The GeneXpert MTB/RIF assay is endorsed as the test of choice for pulmonary TB and has demonstrated promising performance for extrapulmonary TB [11]. It provides results within 2 hours and detects rifampicin resistance, facilitating early initiation of standard anti-TB therapy. However, it cannot distinguish between live and dead bacilli and may yield false-positive results due to DNA contamination [5]. Moreover, it is validated by the WHO only for sputum samples [6]. Extrapulmonary applications, such as synovial fluid and bone tissue, are not formally validated and require local validation [6]. Therefore, PCR-based methods such as GeneXpert are not a substitute for culture but serve as complementary tools for rapid and accurate TB diagnosis. In practice, molecular testing can be performed as soon as clinical suspicion arises, particularly when imaging and clinical features are suggestive, allowing early initiation of therapy while awaiting culture or histopathology results. This approach helps reduce diagnostic delays and improves patient outcomes.

## Conclusion

This report describes a case of a 29-year-old immunocompetent male in whom molecular testing revealed OA TB with asymptomatic pulmonary involvement, allowing the initiation of standard anti-tuberculous therapy and resulting in favourable clinical and functional outcomes.

This case highlights the importance of considering OA TB in the differential diagnosis of chronic joint swelling, even in immunocompetent patients, particularly in endemic regions or among exposed individuals.

Early molecular testing is key to reducing diagnostic delays and preventing complications, such as joint destruction, despite its limitations.

Furthermore, evaluation for associated pulmonary TB should be systematically performed, even in the absence of suggestive clinical signs.

## Abbreviations

CT: computed tomography
HIV: human immunodeficiency virus
MRI: magnetic resonance imaging
MTB/RIF: *Mycobacterium tuberculosis*/Rifampicin
OA TB: osteoarticular tuberculosis
TB: tuberculosis
WHO: World Health Organization
